# Robotic versus open radical Prostatectomy: comparing automobiles and carriages in 2024

**DOI:** 10.1590/S1677-5538.IBJU.2024.0470

**Published:** 2024-08-25

**Authors:** Tomás Bernardo Costa Moretti, Leonardo Oliveira Reis

**Affiliations:** 1 Pontifícia Universidade Católica de São Paulo Departamento de Urologia São Pailo SP Brasil Departamento de Urologia, Pontifícia Universidade Católica de São Paulo, PUC-São Paulo, São Pailo, SP, Brasil;; 2 Universidade de Campinas Campinas SP Brasil UroScience, Universidade de Campinas, UNICAMP, Campinas, SP, Brasil;; 3 Pontifícia Universidade Católica de Campinas Campinas SP Brasil ImunOncologia, Pontifícia Universidade Católica de Campinas, PUC-Campinas, Campinas, SP, Brasil

## COMMENT

The second Randomized Clinical Trial (RCT) to date comparing robotic (RARP) versus open (RRP) radical prostatectomy, the São Paulo trial (1) highlights the challenges of randomization in a period where technological access is widespread in the US and Europe. The Brisbane Trial, published 8 years ago, stands as the first and only comparator in this context (2).

As new scientific insights emerge from centers adopting robotic surgery, RARP is increasingly viewed as the gold standard in current technology. However, open surgery can provide comparable oncological control and late quality of life and remains prevalent in developing countries due to limited resources. While robotic surgery may offer slightly better early sexual and urinary function, these remain secondary outcomes in both RCTs conducted so far.

In the hierarchy of evidence, systematic reviews and RCTs are deemed the most robust. To delineate the natural history of Radical Prostatectomy (RP), the Reverse Systematic Review (RSR) method, recently described by Moretti TBC and Reis LO, compiled a population-based database named EVIDENCE. This database amalgamates data from 910 studies across 80 Systematic Reviews (SR) on RRP, laparoscopic, and RARP, encompassing 1,353,485 patients (3–8). The clinical heterogeneity generated by RSR allows EVIDENCE to provide central tendency values for population samples with a narrow standard error of the mean, enhancing the precision of mean values relative to the population. This heterogeneity also increases the generalization and representativeness, serving as a practical reference for urologists in real-world settings, and enabling comparisons across the available RCT.

Table-1 summarizes key outcomes comparing the EVIDENCE database (3–6), São Paulo Trial [1], and the Brisbane Trial [2] and presents a didactic graphic representation for the pentafecta results between open and robotic radical prostatectomy by different assays. Values are color-coded (significant difference - red for above, green for below - and yellow for non-significant difference). While it is noted that the EVIDENCE was able to predict the results of the RTC's, acting as a weighting factor for the averages through its representative heterogeneity of scenarios, the São Paulo Trial [1] tends to report higher values, while the Brisbane Trial [2] reports lower values compared to EVIDENCE, illustrating how different randomized studies can depict diverse scenarios that require careful comparison.

**Table 1:** Summarizes key outcomes comparing the EVIDENCE database.

		Moretti TBC et al. (3–7) (EVIDENCE Database)				Nahas W et al. (1) (São Paulo RTC)		Coughlin GD et al. (2) (Brisbane RTC)	
**Surgery period**		**Jan, 1962 to Apr, 2018**				**Feb, 2014 to Jul, 2018**		**Aug, 2010 to Nov, 2014**	
**Total n**		**RRP**		**RARP**		**RRP**	**RARP**	**RRP**	**RARP**
		881,719		366,006		156	171	151	157
**Preoperative**		**Mean**	**SE**	**Mean**	**SE**	**CTM**	**CTM**	**CTM**	**CTM**
Age (years)		**62.8**	0.16	**61.4**	0.1	**64.0**	**64.0**	**60.4**	**59.6**
BMI (m\kg/m2)		**26.2**	0.17	**27.0**	0.1	**27.1**	**27.3**	NA	NA
iPSA (ng/ml)		**8.9**	0.26	**7.7**	0.2	**7.9**	**7.2**	**7.6**	**7.4**
**cT (%)**									
	cT1	**58.7**	1.36	**68.7**	1.0	**49.4**	**48.5**	NA	NA
	cT2	**38.7**	1.28	**31.7**	1.0	**46.2**	**45.0**	NA	NA
	cT3	**8.0**	1.24	**5.9**	0.7	**4.5**	**6.4**	NA	NA
**cISUP (%)**									
	1	**55.9**	1.5	**53.2**	1.1	**50.0**	**46.2**	**15.0**	**18.0**
	2 3	**34.2**	1,2	**35.6**	0.8	**30.1** **9.6**	**33.3** **10.5**	**50.0** **18.0**	**45.0** **22.0**
	4 5	**11.1**	0.9	**12.6**	0.8	**7.7** **2.6**	**6.4** **3.5**	**7.0** **10.0**	**9.0** **6.0**
**Surgical**		**Mean**	**SE**	**Mean**	**SE**	**CTM**	**CTM**	**CTM**	**CTM**
Operative Time (min)		**169.5**	3.9	**199.8**	3.0	**120.0**	**212.0**	**234.3**	**202.0**
EBL (mL)		**852.1**	29.9	**228.2**	6.2	**719.5**	**250.0**	**1338.1**	**443.7**
Blood Transfusion (%)		**19.8**	1.5	**2.8**	0.3	**1.3**	**0.0**	**2.0**	**1.0**
Complication (%)		**20.2**	1.4	**12.3**	0.5	**17.3**	**11.1**	**9.0**	**4.0**
**Oncological**		**Mean**	**SE**	**Mean**	**SE**	**CTM**	**CTM**	**CTM**	**CTM**
**pT (%)**									
	pT2	**66.9**	1.0	**73.6**	0.7	**60.9**	**50.9**	**68.0**	**65.0**
	pT3	**31.6**	1.0	**25.9**	0.8	**39.1**	**49.1**	**31.0**	**35.0**
	pT3a	**22.3**	0.9	**18.6**	0.7	**29.5**	**39.1**	**28.0**	**29.0**
	pT3b	**10.3**	0.8	**7.2**	0.5	**9.6**	**10.0**	**5.0**	**6.0**
**pISUP (%)**								
	1	**44.3**	1.6	**36.3**	1.0	**13.5**	**13.5**	**3.0**	**4.0**
	2 3	**45.1**	1.3	**52.4**	0.9	**62.8** **15.4**	**56.7** **17.5**	**48.0** **38.0**	**46.0** **40.0**
	4 5	**13.2**	1.0	**10.8**	0.6	**2.6** **5.8**	**4.1** **8.2**	**0.0** **11.0**	**1.0** **9.0**
**PSM (%)**									
	Total	**23.6**	0.7	**19.7**	0.5	**29.5**	**36.3**	**10.0**	**15.0**
	pT2	**13.3**	0.9	**11.7**	0.6	**22.1**	**25.3**	**2.0**	**3.0**
	pT3	**44.3**	2.2	**40.5**	1.3	**41.0**	**47.6**	**8.0**	**11.0**
Biochemical Reccurence (%)		5 years				3 years		2 years	
		**20.4**	13.3	**23.4**	12.0	**16.0**	**24.0**	**9.0**	**3.0**
**Functional**		**Mean**	**SE**	**Mean**	**SE**	**CTM**	**CTM**	**CTM**	**CTM**
Continence (%)		0-1 PAD				0-1 PAD		0-1 PAD	
	3 months	**63.8**	0.7	**74.7**	0.1	**64.7**	**80.5**	NA	NA
	6 months	**78.7**	0.5	**84.8**	0.1	**81.6**	**90.1**	**87.0**	**87.0**
	12 months	**91.0**	0.1	**91.0**	0.1	**83.8**	**90.4**	**93.0**	**90.0**
	18 months	**93.0**	0.1	**93.0**	0.1	**78.8**	**95.4**	NA	NA
Potency (%)		SHIM ≥ 17				SHIM ≥ 17		ESI ≥ 50%	
	3 months	**30.0**	0.4	**23.8**	1.4	**5.3**	**23.9**	NA	NA
	6 months	**43.5**	0.5	**51.1**	1.2	**6.9**	**30.6**	**22.0**	**22.0**
	12 months	**24.8**	0.4	**35.0**	0.3	**24.0**	**37.8**	**30.0**	**35.0**
	18 months	NA	NA	**59.0**	0.1	29.6	**39.8**	NA	NA

**Legend:** RRP = Retropubic Radical Prostatectomy; RARP = Robot-assisted Radical Prostatectomy; n = number of patients; CTM = Central Tendency Measure (mean or median); SE = Standard Error; BMI = Body Mass Index; iPSA = initial Prostate Specific Antigen; cT = clinical T Stage; cISUP = clinical ISUP Grade group; EBL = Estimated Blood Loss; pT = pathological T Stage; pISUP = pathological ISUP Grade group; PSM = Positive Surgical Margins; SHIM = Sexual Health Inventory for Men; ESI = Erections Sufficient for Intercourse more than 50% of the time; NA = Not available. Values in bold: Black = EVIDENCE reference (95% Confidence interval +- 2 x SE); Green - below 95% IC; Red - above 95%IC; Yellow - inside 95% CI.

Surgeon related variabilities might play a significant role in the disagreements illustrated in Table-1, even between São Paulo and Brisbane randomized controlled trials, considering the wide variability among surgeons performing radical prostatectomy. The higher variability of the results in the São Paulo study might be related to the participation of more surgeons, compared to smaller differences between the results in the Brisbane study, carried out by only one surgeon in each technique.

Most surgical trials represent, in a great measure, the comparison of surgeons’ performances, with limited generalizability, also diverse robotic platform systems might implicate in different performance, to be compared in the future (9). Compared to pharmacological trials that utilize identical drugs and doses, surgical randomized trials are unique regarding the inherent diversities related to the human surgeons and the surgical theater. The relevance and accuracy of an RCT's findings depend heavily on the rigor of its design, execution, and analysis. This rigorous process, while essential, might limit the study's reproducibility compared to the representativeness of the RSR (4), mainly regarding the surgical performance due to disparities in skill and experience between surgeons and centers. Advances of generative AI will soon transform surgery in a more predictable science, as technical, ethical and regulatory evolution rapidly evolves to progressive surgical platform autonomy (10), making surgical trials less surgeon dependent, reaching the drug consistency of pharmacological trials.

Considering the existing evidence, the scientific consensus may not support substantial changes regarding the advantages of RARP over RRP. The diversity of scenarios makes the comparison inherently biased, with RRP predominating in developing countries with focus on short term cost-effectiveness, versus RARP concentrated in centers that hold cutting-edge technologies and are responsible for scientific and technological development.

Ultimately, this debate is more about access in public health than declaring a definitive winner, as the truth in this field remains dynamic, subjective, and sometimes contradictory over time. From a pragmatic point of view, much beyond the difficulty of carrying out adequate and representative controlled studies, the scientific community has progressively lost interest in reconciling these two scenarios, which emulate the challenges of comparing automobiles and carriages, different journeys that lead to the same destination.

While studies on cost-effectiveness and quality of life can still influence decisions, particularly in resource-constrained healthcare settings, including those in developing countries, the debate on functional outcomes, mainly in the short and midterm might question the randomization of patients between the two techniques. As advances in surgical techniques, imaging and robotic systems continue to evolve, it is crucial to further refine outcomes and broaden accessibility. Future studies may address disparities across diverse healthcare settings, with efforts focused on expanding access and enhancing habilitation in advanced surgical technologies in a rapidly transforming scenario.

For those less attached to immediateness, intriguingly the RSR identified a lack of RARP data on intermediate and long follow-up (4), compromising the EVIDENCE, and the available RCT are so far limited to 12 months. RARP long term oncological control needs more robust evidence. As technology evolves, the "new" surgical negative margins might not guarantee the results of wider margins of open dissection. If there is an oncological price to be paid for the functional short-term gains is still an open question for the long-term robust evidence.

After over 20 years of scientific debate, thousands of under-analyzed studies, hundreds of them summarized in 80 systematic reviews (EVIDENCE) and two RTC's highlight the complexity of the subject at a global healthcare level. In the new era of big data, perhaps it is time for the scientific community to explore new ways of exploring data, through connecting real-life data in a multicentric and real-time way with the implementation of data-driven culture and business intelligence tools, capable of showing the beauty of a realistic heterogeneity.
